# Elevated baseline C-reactive protein levels predict poor progression-free survival in sporadic vestibular schwannoma

**DOI:** 10.1007/s11060-021-03918-0

**Published:** 2021-12-09

**Authors:** Johannes Wach, Ági Güresir, Valeri Borger, Patrick Schuss, Albert Becker, Christoph Coch, Marie-Therese Schmitz, Michael Hölzel, Marieta Toma, Ulrich Herrlinger, Hartmut Vatter, Erdem Güresir

**Affiliations:** 1 Department of Neurosurgery, Rheinische Friedrich-Wilhelms-University, Venusberg-Campus 1, Bonn, Germany; 2 Department of Neuropathology, Rheinische Friedrich-Wilhelms-University, Bonn, Germany; 3 Institute of Clinical Chemistry and Clinical Pharmacology, Rheinische Friedrich-Wilhelms-University, Bonn, Germany; 4 Department of Medical Biometry, Informatics and Epidemiology, Rheinische Friedrich-Wilhelms-University, Bonn, Germany; 5 Institute of Experimental Oncology, Rheinische Friedrich-Wilhelms-University, Bonn, Germany; 6 Institute of Pathology, Rheinische Friedrich-Wilhelms-University, Bonn, Germany; 7 Division of Clinical Neurooncology, Department of Neurology and Centre of Integrated Oncology, Rheinische Friedrich-Wilhelms-University, Bonn, Germany

**Keywords:** C-reactive protein, Inflammation, Progression, Vestibular schwannoma

## Abstract

**Background:**

Recent investigations showed emerging evidence of the role of inflammation in the growth of sporadic vestibular schwannoma (VS). The present retrospective study investigated the impact of systemic inflammation on tumor progression using serum C-reactive protein (CRP) levels in a series of 87 surgically treated sporadic VS patients.

**Methods:**

The optimal cut-off value for CRP was defined as 3.14 mg/dl according to the receiver operating characteristic curve (AUC: 0.70, 95% CI 0.47–0.92). Patient cohort was dichotomized into normal (n = 66; < 3.14 mg/dl) and high baseline (n = 21; ≥ 3.14 mg/dl) CRP groups.

**Results:**

No significant differences in age, sex, comorbidities influencing the systemic inflammatory state, Karnofsky performance status (KPS), tumor size, extent of resection, or MIB-1 index were identified between the two groups defined by the baseline CRP levels. Univariable analysis demonstrated that a high CRP level (≥ 3.14 mg/dl) is significantly associated with a shortened progression-free survival (PFS) (hazard ratio (HR): 6.05, 95% CI 1.15–31.95, *p* = 0.03). Multivariable Cox regression analysis considering age, extent of resection, KPS, tumor size, and baseline CRP confirmed that an elevated CRP level (≥ 3.14 mg/dl) is an independent predictor of shortened PFS (HR: 7.20, 95% CI 1.08–48.14, *p* = 0.04).

**Conclusions:**

The baseline CRP level thus serves as an independent predictor of PFS. Further investigations of the role of inflammation and tumor inflammatory microenvironment in the prediction of prognosis in sporadic VS are needed.

**Graphical abstract:**

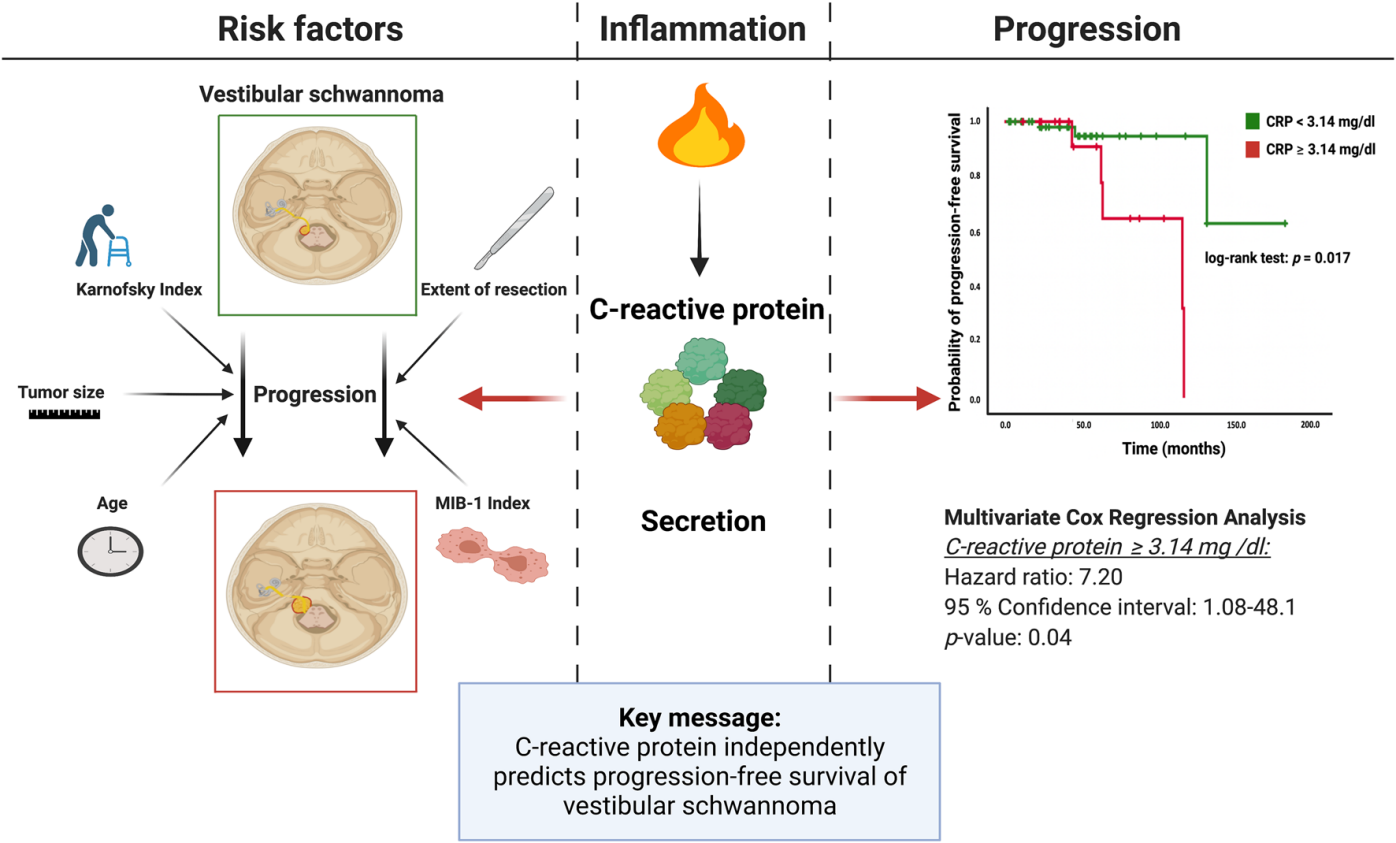

**Supplementary Information:**

The online version contains supplementary material available at 10.1007/s11060-021-03918-0.

## Introduction

Vestibular schwannoma (VS) is a benign tumor that accounts for 75% of all lesions in the cerebellopontine angle, and it originates from the Schwann cells covering the eighth cranial nerve [1].

It has been suggested that complete resection is the optimal treatment to achieve long-term tumor control in giant VS [2]. However, data also suggest that subtotal resection with subsequent irradiation of tumor remnants, if necessary, is also a feasible strategy to achieve a nearly similar results regarding progression-free survival (PFS) [3, 4]. Furthermore, systematic reviews have confirmed that stereotactic radiosurgery achieves sufficient long-term tumor control and hearing preservation for newly diagnosed small VS [5]. In general, a stringent imaging follow-up using magnetic resonance imaging (MRI) of residual tumor tissues is recommended for at least 7–10 years [6].

Systemic inflammation is known to be associated with shortened time to tumor progression in patients with cancer [7, 8]. Recent investigations emphasized the role of inflammation in the growth of VS and the functional outcome following surgery for VS [9–11]. Furthermore, it was discovered that cyclooxygenase-2 (COX-2) expression in VS is significantly associated with tumor proliferation and cell growth both in human tumor and in vitro [12, 13]. To date, despite its paramount importance for optimizing patients´ therapy and functional outcome following surgery, no predictive factors for VS growth have been identified [14, 15].

C-reactive protein (CRP) is an inflammation-related prognostic biomarker that can predict tumor recurrence and treatment response in adult solid tumors [16]. However, the predictive value of systemic inflammation based on CRP in VS is unknown so far. The purpose of the present study was to assess the relationship between baseline serum CRP levels at initial diagnosis and progression-free survival in a homogenous population of sporadic VS patients who underwent surgical resection via the retrosigmoid approach without postoperative adjuvant radiation therapies.

## Materials and methods

### Study design

**Fig. 1:**
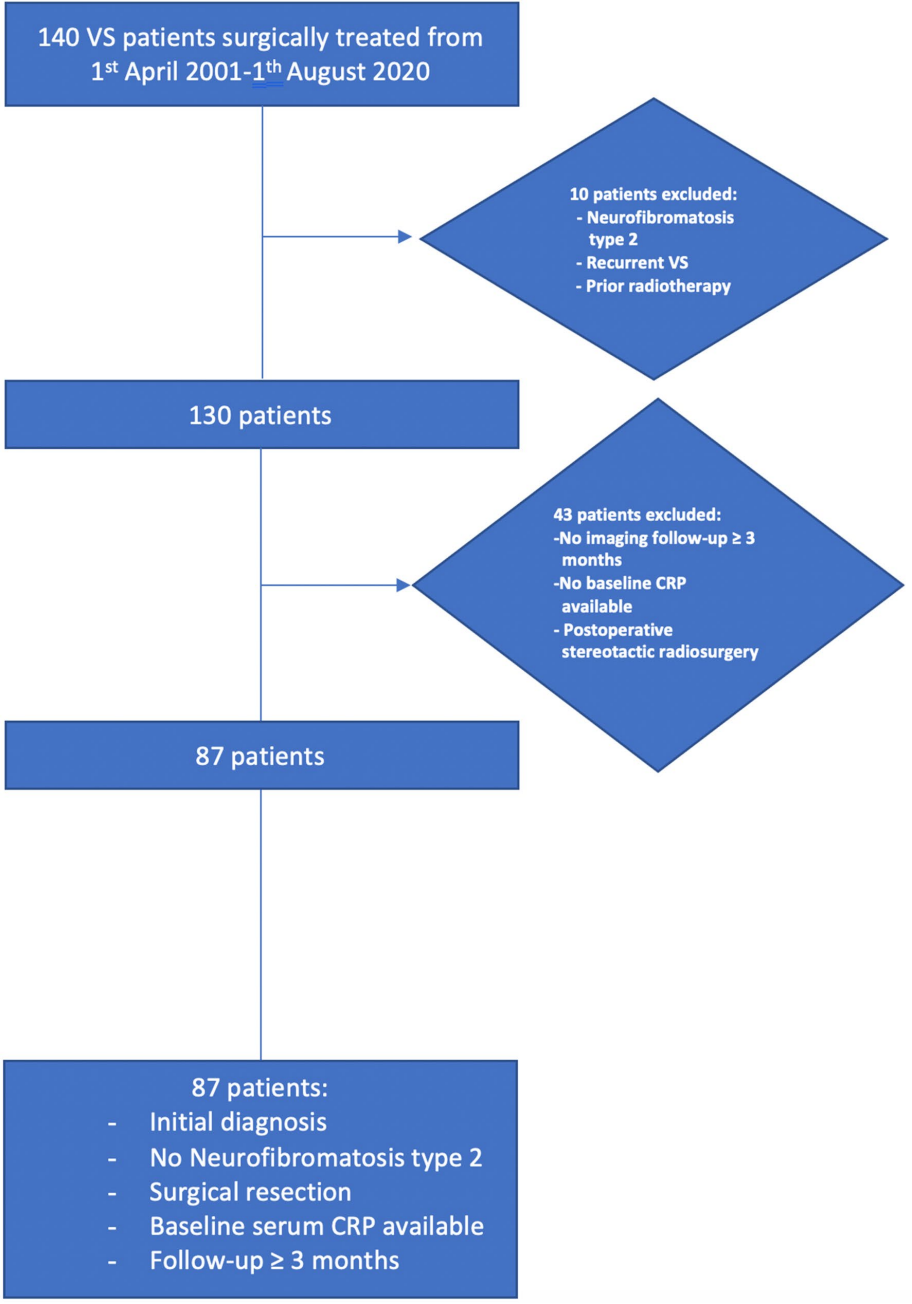
Flow chart illustrating the selection process of consecutive vestibular schwannoma patients between 2001 and 2020

We retrospectively reviewed 140 VS patients from April 2001 to August 2020. Patients with prior radiotherapy, surgery for recurrent VS, stereotactic radiosurgery after resection, neurofibromatosis type 2, lack of imaging follow-up data (≥ 3 months), and lack of baseline CRP levels data were excluded. Figure 1 illustrates the patient selection process, which identified 87 eligible patients. Treatment decision making was based on a multidisciplinary consensus between radiotherapists, otolaryngologists, neuro-oncologists, and neurosurgeons. In the decision-making process, the tumor size, its growth rate, the probability of gross total or near total resection with preservation of hearing and facial nerve function, the patient’s age, patient’s comorbidities, and the patient preferences were considered. Primary radiosurgery or stereotactic radiotherapy was applied for small VSs (< 25 mm in diameter) with an observed tumor growth in the wait-and-scan policy in patients having functional hearing. However, for larger VSs (≥ 25 mm in diameter) with an observed tumor growth in the wait-and-scan policy, VS patients with symptoms of brainstem compression, young (< 70 years) VS (≥ 25 mm) patients with functional hearing loss or functional hearing ability, small VSs (< 25 mm) with an observed tumor growth in patients with a functional hearing loss, and VS patients with occlusive hydrocephalus, we recommend a surgical resection. Primary radiosurgery or stereotactic radiotherapy for tumors with a diameter greater than 25 mm can be problematic due to the potential development of a perilesional edema [17]. This decision algorithm is also supported by several guidelines [18].

### Biochemical measurements

Venous blood samples [examination profile: complete blood count, kidney, liver tests, and the coagulation profile (INR, aPTT)] were routinely collected within 24 h prior to the surgery and data acquisition was performed using Lauris (version 17.06.21, Swisslab GmbH, Berlin, Germany). These laboratory measurements were performed at constant time points, which made it possible to homogeneously analyze time-to-tumor progression. The serum CRP values were obtained by turbidimetric immunoassays with a CRPL3 reagent (Roche, Basel, Switzerland) displaying standard CRP levels. High-sensitivity CRP levels were not included to ensure a homogeneous analysis.

### Surgical technique

All patients underwent tumor resection using the retrosigmoid approach. Surgery was performed in the same workflow as previously reported [11].

### Immunohistochemistry

Surgical specimens were routinely evaluated with haematoxylin/eosin staining and processed for immunohistochemical reactions with antibodies directed against: S-100 (DAKO, Glastrop, Denmark), Ki-67 (MIB1; DAKO, Glastrop, Denmark), and cluster of differentiation (CD) 68 (Clone KP1, dilution 1:1000, DAKO, Glastrop, Denmark). The MIB-1 index was determined in randomly selected high-power microscopic fields in 83 (83/87; 95.4%) patients. The proportions of stained and unstained nuclei in the tumor cells were determined. The MIB-1 index was defined as the percentage of Ki-67^+^ nuclei. Macrophage infiltrates were investigated using CD68 staining.

### Data recording and analysis

The following general preoperative patient characteristics were recorded: age, sex, Karnofsky performance status (KPS), and tumor size.

#### Tumor size

Tumor size was categorized according to the largest extrameatal tumor diameter in the cerebellopontine angle on post-contrast axial T1-weighted MR images. The tumor size in term of the diameter was defined as follows: class 3, > 4 cm; class 2, 2–4 cm; and class, 1 < 2 cm [11, 19].

#### Extent of resection

GTR was defined as complete resection without any residual nodular enhancement, whereas STR was defined as an extent of resection of 90–99% of the tumor [11, 20]. The extent of resection was assessed by independent neuroradiologists using pre- and postoperative 1.5 or 3.0 Tesla MR images. First, follow-up MR images were performed three months after surgery and further examinations were executed on an annual basis.

#### Tumor progression

Tumor progression was defined as a tumor growth with an increase of > 20% over the previous tumor volume or a size 2 mm larger than that found on the previous MRI and the intention to treat the tumor [11, 21, 22].

### Statistics

Data were analyzed using SPSS© version 27.0 (IBM Corp, Armonk, New York, USA). Receiver-operating characteristic (ROC) curves were constructed and the area under the receiver-operating characteristic curve (AUC) for CRP in the prediction of VS progression was determined. Furthermore, optimal cutoff point of CRP for dichotomization of this continuous variable was defined using the minimum *p*-value approach, which displays the log-rank test results regarding PFS [23]. Time-dependent ROC curve was created to evaluate the predictive value of serum CRP for PFS after 5-, and 10 years using the R software v4.0.4 (R Foundation for Statistical Computing, Vienna, Austria) and the R package “risksetROC” [24]. Frequency distribution histogram of serum CRP values was also created. Univariable analyses of the proportions of categorical data among the CRP groups were performed using Fisher´s exact test (two-sided). A *p*-value < 0.05 was defined as statistically significant. Furthermore, a uni- and multivariable Cox regression analysis of PFS was performed. Age, KPS, and Tumor size were included in the multivariable Cox regression analysis because those items are potential confounding variables and might be related to extent of resection.

## Results

### Patient characteristics

A total of 87 consecutive VS patients were analyzed. Mean age (± Standard deviation [SD]) was 53.6 (± 15.1) years and there was a female predominance (female:male = 1.18:1). Fifteen (17.2%), 63 (72.5%), and 9 (10.3%) patients had a tumor defined as tumor size class 1, 2, and 3, respectively. The median (25th–75th percentile) KPS was 90.0 (80–90). Gross total resection (GTR) was achieved in 58 (66.7%) patients, whereas subtotal resection (STR) was performed in 29 (33.3%) patients. The mean (± SD) Molecular Immunology Borstel (MIB)-1 index was 4.18 (± 1.35).

### CRP and cut-off determination

**Fig. 2:**
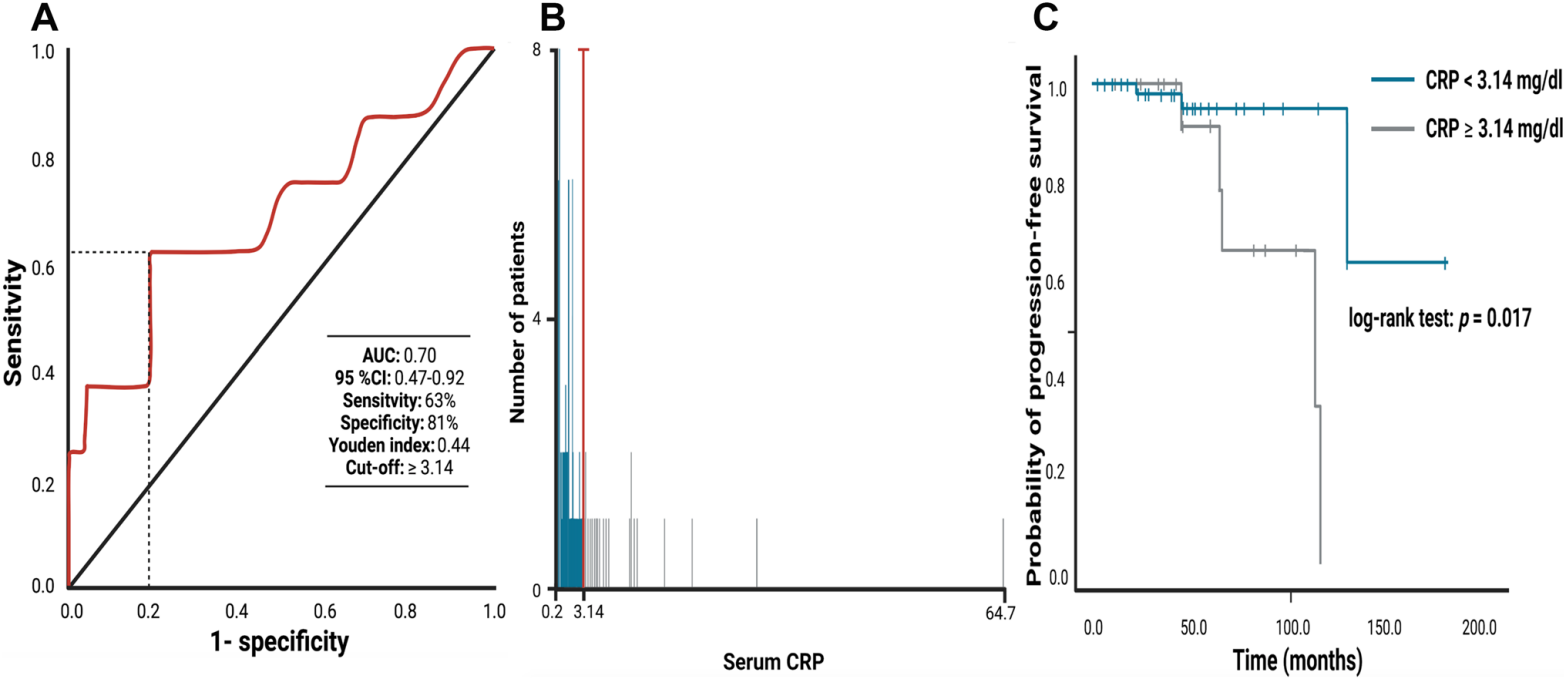
**A** Receiver-operating characteristic curve illustrating the ability of baseline serum CRP level to predict tumor progression of vestibular schwannoma. The area under the ROC curve (AUC) of baseline CRP level for tumor progression was 0.70 [95% confidence interval (CI) 0.47–0.92]. Sensitivity and specificity of baseline CRP level for predicting tumor progression was 63.0% and 81.0%, respectively, with a threshold of ≥ 3.14 mg/dl. **B** Frequency distribution histogram for serum CRP levels in the analyzed cohort. Blue and grey bars indicate the number of patients exhibiting normal and high serum CRP levels, respectively. The red line at the junction of two colors displays the optimized cut-off point for serum CRP (CRP < / ≥ 3.14 mg/dl). **C** Kaplan–Meier analysis of the tumor progression probability stratified by “normal” CRP (< 3.14 mg/dl) and “high” CRP (≥ 3.14 mg/dl). The blue line represents the group of patients with normal CRP levels, whereas the grey line represents the group of patients with elevated CRP levels. Vertical dashes indicate censored data (here: progression-free at last follow-up) in the progression-free survival curves. The time axis is right-censored at 200 months. *p* = 0.017 (log-rank test)

The mean (range) of baseline serum CRP levels was 3.31 mg/dl (0.2–64.7). A ROC curve was constructed to determine a CRP cut-off value in the prediction of tumor progression. The AUC of baseline serum CRP for tumor progression was 0.70 (95% Confidence interval (CI) 0.47–0.92; Youden index: 0.44; Fig. 2A). Sensitivity and specificity of baseline CRP level for predicting recurrence was 63% and 81%, respectively, with a threshold of ≥ 3.14 mg/dl. Time-dependent AUCs of baseline serum CRP for 5-year, and 10-year PFS were 0.70 and 0.69, respectively. The C-index was 0.63 (95% CI 0.52–0.80) and indicates the probability that the baseline serum CRP value in a patient with a shorter time to tumor progression after surgery is higher compared to subjects with no or longer time to progression. Furthermore, patients were dichotomized into normal (< 3.14 mg/dl) and high (≥ 3.14 mg/dl) CRP groups according to the most significant split in the log-rank test (*p* = 0.017) regarding PFS (Supplementary Table 1). Figure 2B displays the optimal cut-point (red line) and the corresponding frequency distribution histogram for serum CRP levels in the cohort. Both methods revealed the same optimal CRP cut-point (< / ≥ 3.14 mg/dl).

### Patient characteristics of CRP groups

The patient cohort was dichotomized into patients with normal (< 3.14 mg/dl) and high (≥ 3.14 mg/dl) baseline CRP levels. Sixty-six (75.9%) patients were allocated to the “normal” CRP group and 21 (24.1%) to the “high” CRP group. Univariable analyses using the Fisher´s exact test (two-sided) were performed regarding distribution of sex (female/male), age (< 60/ ≥ 60), KPS (< 90/ ≥ 90), tumor size class (< 4 cm/ ≥ 4 cm), extent of resection (GTR/STR), and the MIB-1 index (< 5%/ ≥ 5%). Patients were also screened for underlying conditions which could induce chronic inflammation and influence serum CRP levels. The incidence of smoking, obesity, diabetes mellitus and connective tissue diseases were homogeneously distributed in both arms. Patients with blood-borne infectious diseases (e.g., HIV, Hepatitis B, C) or with a malignant cancer were not present in both groups. Potential confounding variables were homogeneously distributed among the CRP groups. Further details are summarized in Table 1.

**Table 1:** Patient characteristics and univariate analysis in normal and high CRP level groups (using Fisher’s exact test (two-sided) & independent t-test)

Characteristics	CRP (n = 87)		
	Normal (< 3.14 mg/dl) (n = 66)	High (≥ 3.14 mg/dl) (n = 21)	*p*-value
Sex (female/male)	34/32	13/8	0.46
Age (< 60 / ≥ 60)	43/23	10/11	0.62
Smoking (≥ 10 cigarettes/d) (yes/no)	16/50	2/19	0.22
Obesity (BMI ≥ 30.0) (yes/no)	18/48	6/15	0.99
Connective tissue diseases (yes/no)	2/64	0/21	0.99
Diabetes mellitus (yes/no)	2/64	0/21	0.99
Karnofsky performance status (< 90/ ≥ 90)	18/48	6/15	0.99
Largest extrameatal tumor diameter (mean ± SD), mm	28.16 ± 9.49	29.26 ± 10.92	0.66
Tumor size classes (1&2/3)	62/4	18/3	0.35
Extent of resection (GTR/STR)	44/22	14/7	0.99
MIB-1 labeling index (< 5%/ ≥ 5%) [available in 83 patients]	38/27	13/5	0.41

*BMI* body mass index; *CRP* C-reactive protein; *GTR* gross total resection; *MIB* molecular immunology borstel; *SD* standard deviation; *STR* subtotal resection

### CRP and macrophages

**Fig. 3:**
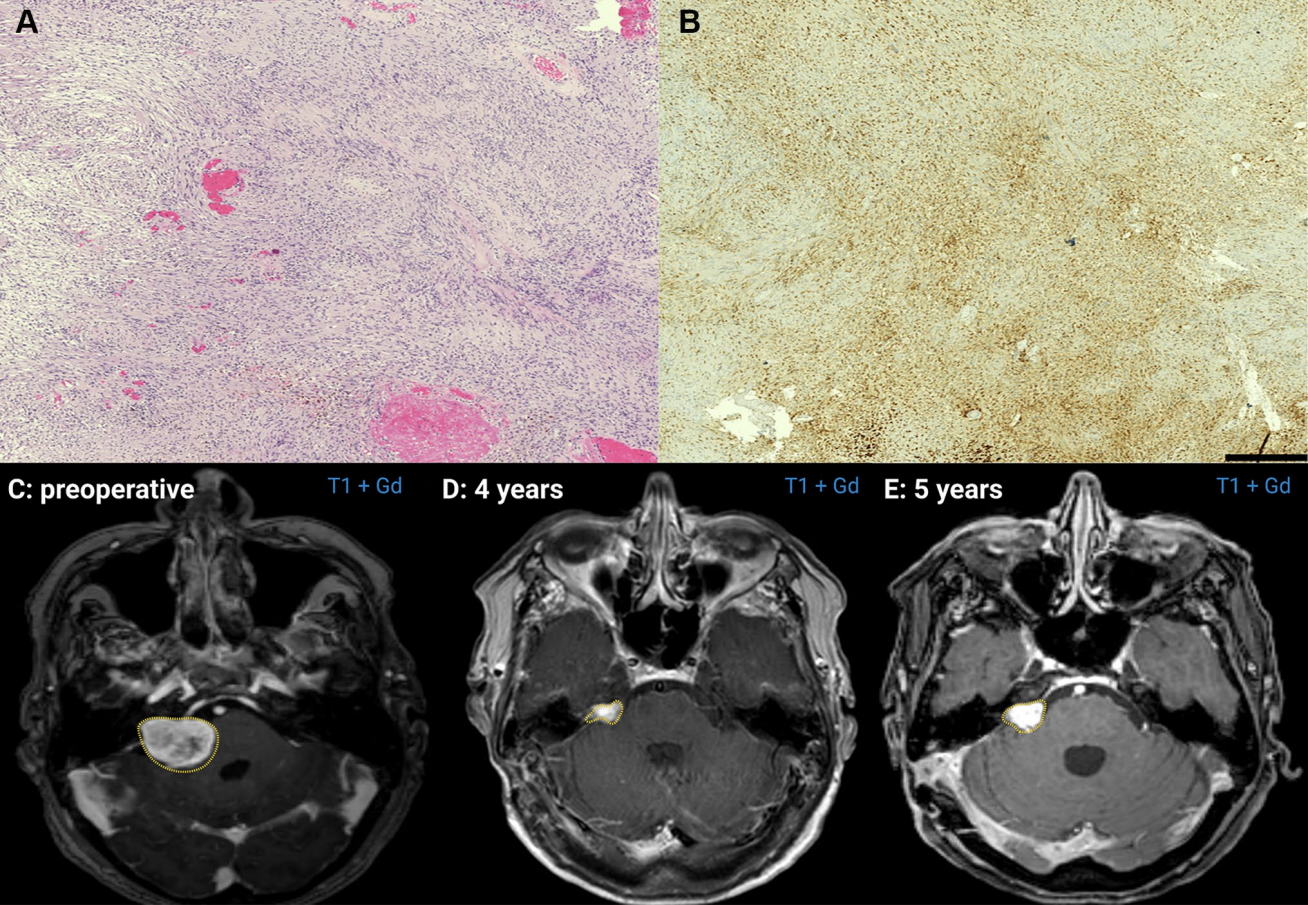
**A** Representative neuropathology of a vestibular schwannoma (**A** hematoxylin & eosin (H&E)) showing dense infiltrates of CD68-positive macrophages (**B** CD68; Clone KP1, dilution 1:1000, DAKO, Glastrop, Denmark; bar graph—500 µm). **C** Preoperative Gadolinium (Gd)-enhanced T1-weighted axial MR-image shows a right-sided giant vestibular schwannoma (volume: 16.2 cm^3^) of the same patient as in the neuropathological figures **D** Gd-enhanced T1-weighted axial MR-image shows a residual tumor at 4 years after subtotal resection of the right-sided giant vestibular schwannoma (volume: 1.5 cm^3^) **E** Gd-enhanced T1-weighted axial MR-image reveals a progress of the residual tumor at 5 years after surgery (volume: 2.0 cm^3^). Volumetric tumor measurements were performed using Brainlab iPlan® software (Brainlab, Feldkirchen, Germany)

CD68 and CD 45 staining was performed in 48 and 14 cases, respectively. Positive staining of macrophages by CD68 was observed in 91.7% (11/12) of patients with a CRP ≥ 3.14 mg/dl and 72.2% (26/36) of those with a CRP < 3.14 mg/dl (Spearman’s rank correlation coefficient: *r* = 0.385, *p* = 0.007). Figures 3A, B show representative histopathological images of CD68-positive macrophage infiltrates within the areas of the tumor in a patient who had a recurrence at 5-years after surgery for VS.

### Influence of baseline serum CRP levels on progression-free survival

The mean (range) of imaging follow-up period in the entire cohort was 46.6 (3.0–183.0) months. Eight (9.2%) patients with tumor progression were identified in the entire cohort. Three (3/66; 4.5%) patients with VS progressions were in the normal CRP level group, whereas 5 (5/21; 23.8%) were in the high CRP level group. Figures 3C–E display a representative case with gadolinium-enhanced T1-weighted magnetic resonance images revealing a progression of a subtotally resected VS. The mean time of progression-free survival in the entire cohort was 132.5 (95% CI 106.5–158.4) months. Patients with a normal baseline CRP level [< 3.14 mg/dl (n = 21)] had a mean PFS of 159.2 months (95% CI 131.8–186.6), whereas patients with an elevated CRP level [≥ 3.14 mg/dl (n = 66)] had a mean PFS of 96.1 months (95% CI 75.1–117.2), respectively (log-rank test: *p* = 0.017; Kaplan–Meier curve: Fig. 2C).

### Uni- and multivariable Cox regression analysis of progression-free survival

A univariable Cox regression analysis of PFS was performed for potential predictors of PFS in VS. Subtotal resection and elevated baseline serum CRP (≥ 3.14 mg/dl) were significantly associated with shortened PFS. We conducted a multivariable Cox regression analysis of PFS considering age (< 60/ ≥ 60), KPS (< 90/ ≥ 90), tumor size (< 4 cm/ ≥ 4 cm), extent of resection (GTR/STR), and baseline CRP (< 3.14/ ≥ 3.14). The multivariable Cox regression analysis identified an elevated baseline serum CRP (≥ 3.14 mg/dl) as a significant and an independent predictor of shortened PFS (Hazard ratio (HR): 7.20, 95% CI 1.08–48.14, *p* = 0.04). Table 2 summarizes the uni- and multivariable Cox regression analyses of PFS in VS.

**Table 2:** Uni- and multivariate Cox regression analysis of progression-free survival in vestibular schwannoma

Variable	Univariate			Multivariate		
	HR	95% CI	*p*-value	HR	95% CI	*p*-value
Age (< 60 vs. ≥ 60)	2.47	0.58–10.50	0.22	1.65	0.26–10.33	0.59
CRP (< 3.14 mg/dl vs. ≥ 3.14 mg/dl)	6.05	1.15–31.95	0.03	7.20	1.08–48.1	0.04
Tumor size classes (small & medium vs. large)	3.79	0.71–20.08	0.12	2.23	0.14–36.65	0.57
Extent of resection (STR vs. GTR)	4.90	1.13–21.18	0.03	6.71	0.85–52.8	0.07
Karnofsky performance status (< 90 vs. ≥ 90)	1.04	0.23—4.66	0.96	1.22	0.18–8.09	0.84
Sex (female vs. male)	1.76	0.40–7.67	0.45			
MIB-1 labeling index (< 5% vs. ≥ 5%)	2.24	0.49–10.18	0.30			

*CI* confidence interval; *CRP* C-reactive protein; *GTR* gross total resection; *HR* hazard ratio; *MIB* molecular immunology borstel; *STR* subtotal resection

## Discussion

In the present study, we evaluated baseline C-reactive protein levels as a biomarker for progression in sporadic VS patients. We observed significantly decreased progression-free survival in patients with a high baseline serum CRP (≥ 3.14 mg/dl) level at the time of VS diagnosis. An elevated CRP level seems to be an independent risk factor for shortened time to tumor progression. Our findings suggest the potential importance of assessing the prognosis of VS by combining clinicopathologic characteristics with the initial systemic inflammatory status.

The rate of progression was 9.2% in our cohort with a mean (range) follow-up period of 46.6 (3.0–183.0) months. This rate is consistent with previous studies that reported rates of recurrence of 8.8% or 10.8% for gross- or subtotally resected VSs [4, 25]. In our cohort, 24.1% of patients presented with elevated CRP serum concentrations, indicating that increased circulating concentrations of classical inflammatory response elements such as CRP are common among VS patients. The patients who had increased baseline levels of CRP had a significantly shorter time-to-tumor progression than those with CRP levels within the normal range (< 3.14 mg/dl). Lewis et al. [9] suggested that inflammation is a paramount parameter when evaluating the growth of VS. Tumor tissues from eight sporadic VS patients were immunohistochemically investigated and the growing tumors exhibited significantly higher proportions of Ki-67/Iba1 (ionized calcium-binding adapter molecule)-positive cells and macrophages in their study. This finding was consistent with our previously reported observation that there is a high proportion of CD68^+^ macrophages accounting for proliferating cells within VSs [11]. Furthermore, it is known that pro-inflammatory cytokines such as transforming growth factor β1, interleukin-1β and interleukin-6 are significantly overexpressed in human VS compared to normal vestibular nerve samples. Neoplastic Schwann cells were found to produce pro-inflammatory cytokines that might act in an autocrine manner, stimulating cellular proliferation [26]. CRP is a major acute-phase reactant and systemic inflammatory parameter, that is derived mainly from hepatocytes in response to interleukin-6 and is then secreted into the systemic circulation [27]. Furthermore, CRP was revealed to play a major role in promoting the differentiation of human monocytes towards a proinflammatory classical M1 macrophage [28]. An immunohistochemical analysis of the expression of CD163 and programmed cell death 1 ligand 1 (PD-L1) in 46 sporadic radiation-naïve and subtotally resected VSs elucidated that VS that progressed after surgical resection had higher numbers of M1 macrophages [29]. The increased expression of PD-L1 in progressive VSs was suggested to be the mechanism resulting in the deactivation of tumor-associated macrophages (TAM), growth of the tumor, and the further infiltration of anti-tumor immune cells. Furthermore, paracrine cellular communication between TAMs and Schwann cells seems to explain the decreased anti-tumor effect of TAMs [29]. Additionally, it was demonstrated that intratumoral macrophages contribute significantly to the production of vascular-endothelial-growth-factor (VEGF), which promotes vasodilatation, increases vascular permeability, and induces angiogenesis [30, 31]. VEGF can also act as a chemoattractant for circulating VEGFR-1-expressing macrophages and induces an inflammatory microenvironment [32–35]. Concerning pathophysiology, increased concentrations of CRP might be induced by the autocrine secretion of interleukin-6 in neoplastic Schwann cells, and CRP promotes the differentiation of monocytes toward proinflammatory M1 macrophages, which are predominant in VS tissue and drive the growth of the tumor through paracrine communication between Schwann cells and TAMs. This potential pathophysiological pathway might be associated with our finding that the baseline serum CRP levels significantly correlated with the proportion of CD68^+^ macrophages.

The baseline determination of serum CRP seems to be useful to identify a subgroup of patients who might be predisposed to early tumor progression. There are some potential implications for future investigations and the clinical care of those patients. Lewis et al. [9] showed that growing VSs that express the PK11195 target translocator protein in macrophages within the tumor tissue can be detected by PET imaging using the tracer ^11^C-(R)-PK11195. Therefore, patients presenting with increased serum levels of CRP might benefit from preoperative or follow-up PET imaging examinations to predict tumor progression and set individualized follow-up imaging intervals. In addition to the diagnostic potential of using PET imaging for VS patients with increased CRP concentrations, future therapeutic approaches for an individualized therapy might benefit from an inexpensive and fast identification of a subgroup of patients who are at risk of tumor progression due to the inflammatory microenvironment via a baseline CRP determination. To date, the anti-VEGF antibody bevacizumab is the only targeted molecular treatment in clinical use for VSs [36, 37]. However, its application and use are only proven for neurofibromatosis type 2, and recent concerns have been raised regarding the use of bevacizumab due to potential cardiovascular and renal complications [38, 39].

Moreover, future trials have to elucidate if those patients having increased serum CRP levels will benefit from early stereotactic radiosurgery in case of residual or progressive tumor. This potential clinical implication has not been addressed so far. Lewis et al. [40] investigated the changes of the vestibular schwannoma microenvironment in patients who underwent stereotactic radiosurgery using multinuclear MRI. They suggested that patients who showed a well response to stereotactic radiosurgery and tumor growth attenuation following stereotactic radiosurgery had a reduced vascular supply of the vestibular schwannoma and a reduced amount of pro-tumoral TAM infiltrates in the tumor microenvironment. Hence, this interesting hypothesis might emphasize that patients with an increased serum CRP in combination with a high density of macrophage infiltrates might benefit from an early stereotactic radiosurgery due to the reduction of vascular supply and decreased infiltration of pro-tumoral TAMs. However, future trials will have to investigate this hypothesis in a thorough study design using imaging methods such as dynamic contrast-enhanced (DCE)-MRI or PET tracers reflecting the inflammatory burden in the absence of resected tissue for histopathological analysis during or after adjuvant stereotactic radiosurgery [9, 10].

COX-2 expression was found to be significantly associated with higher MIB-1 expression [12]. COX-2-inhibiting drugs such as salicylates can be a potential treatment option for VS patients with increased systemic inflammatory parameters. In vitro and clinical findings suggested that aspirin has a cytostatic effect, and VS patients taking aspirin demonstrated reduced tumor growth [13]. In contrast, a retrospective analysis of 1048 patients showed that the intake of acetylsalicylic acid, non-steroidal anti-inflammatory drugs, glucocorticoids and other immunosuppressants did not result in differences in COX-2 or MIB-1 expression [12]. However, it is unlikely that the dosage of 100 mg, which is used in Germany and many other countries, can sustain an antiplatelet effect and has an impact on COX-2 because the antiproliferative regulation is based on a transcriptional level and is not regulated by the acetylation of the amino acid serine residue within the enzyme COX-2 [41, 42]. An ongoing prospective phase II, double-blind longitudinal and randomized trial is currently recruiting VS patients to investigate aspirin (administered 325 mg twice daily) intake regarding the tumor growth inhibition (clinical trial number: NCT03079999). This trial might clarify the potential role of acetylsalicylic acid as an antiproliferative treatment option for sporadic VS patients.

## Limitations

The retrospective analysis of clinical data is the major limitation of the present study. Moreover, during this study, which investigated patients in a time period from 2001 to 2020, MRI quality improved significantly, which might have influenced the analysis of PFS. However, we applied highly selective inclusion criteria, such as the analysis of patients who have sporadic VSs, underwent surgical resection for VS at initial diagnosis only, and did not receive any kind of radiation therapy before or after surgery, to achieve a reliable analysis in a homogeneous cohort. Furthermore, baseline CRP values, histopathological reports, and imaging data at presentation and follow-up, which were used as the main data records were extracted from a computerized hospital information system and therefore not affected by the retrospective design.

## Conclusions

Baseline serum CRP level is a potential prognostic marker that can independently predict progression-free survival in VS. Our investigation emphasizes the need for further studies evaluating the role of inflammation in the prognosis of VS.

## Supplementary Information

Below is the link to the electronic supplementary material.

Supplementary file1 (DOCX 16 KB)

## Data Availability

Research data will not be shared.

## Abbreviations

AUC: Area under the receiver-operating characteristic curve
CD: Cluster of differentiation
CI: Confidence interval
COX-2: Cyclooxygenase-2
CRP: C-reactive protein
CSF: Cerebrospinal fluid
DCE: Dynamic contrast-enhanced
GTR: Gross total resection
HR: Hazard ratio
KPS: Karnofsky performance status
MIB: Molecular immunology borstel
MRI: Magnetic resonance imaging
PD-L1: Programmed cell death 1 ligand 1
PFS: Progression-free survival
ROC: Receiver-operating characteristic (ROC)
STR: Subtotal resection
TAM: Tumor-associated macrophage
VS: Vestibular schwannoma
