# Supplementary material for: Elevated baseline C-reactive protein levels predict poor progression-free survival in sporadic vestibular schwannoma
Source: J Neurooncol. 2021 Dec 9;156(2):365–75. doi: 10.1007/s11060-021-03918-0 (PMC8816751; doi:10.1007/s11060-021-03918-0)
Supplement: Supplementary file 1 — Supplementary file1 (DOCX 16 KB) [file 11060_2021_3918_MOESM1_ESM.docx]

**Supplementary Material**

**Supplementary Table 1** Cutoff points of serum C-reactive protein for dichotomization using log-rank test split regarding the progression of vestibular schwannoma

**Supplementary Table 1** Cutoff points of serum C-reactive protein for dichotomization using log-rank test split regarding the progression of vestibular schwannoma

| Cut-Off (mg/dl) | Number (Normal-/High-CRP Group) | *p*-Value |
| --- | --- | --- |
| </≥ 2.47 | 60/27 | 0.18 |
| </≥ 2.55 | 61/26 | 0.16 |
| </≥ 2.7 | 62/25 | 0.16 |
| </≥ 2.85 | 63/24 | *0.022* |
| </≥ 2.95 | 64/23 | *0.021* |
| </≥ 3.04 | 65/22 | *0.018* |
| **</≥ 3.14** | **66/21** | ***0.017*** |
| </≥ 3.30 | 67/20 | 0.11 |
| </≥ 3.45 | 68/19 | 0.37 |
| </≥ 3.75 | 69/18 | 0.34 |
